# The midterm outcome and MACE of robotically enhanced grafting of left anterior descending artery with left internal mammary artery

**DOI:** 10.1186/1749-8090-9-19

**Published:** 2014-01-17

**Authors:** Roberto Casula, Espeed Khoshbin, Thanos Athanasiou

**Affiliations:** 1 Department of Cardiothoracic Surgery, Hammersmith Hospital, Shepherds Bush, London, England W12 0HS, UK; 2 Espeed Khoshbin, University Hospital of Central Manchester, Oxford Road, Manchester M13 9WL, UK; 3 The Department of Biosurgery and Surgical Technology, Imperial College London, 10th Floor, Queen Elizabeth the Queen Mother (QEQM) Building St Mary’s Hospital Campus Praed Street, London W2 1NY, UK

**Keywords:** Da Vinci, Robotic, Coronary artery bypass grafting, MACE

## Abstract

**Background:**

We assessed the midterm outcome and the incidence of major adverse cardiovascular events in UK’s largest Da Vinci assisted robotic coronary revascularisation cohort. This study was set up at the Imperial College NHS Trust, St. Mary’s Hospital, London, United Kingdom.

**Method:**

Benchmarking approach through retrospective audit of the regional outcomes against standards in the published literature. Data was collected from the patient’s records, communication with the primary care physicians and the national strategic tracing service. The results were compared with the published literature. Patients who underwent robotic assisted coronary revascularisation were included. Other robotic procedures or minimally invasive revascularisation without the use of the Da Vinci robot were excluded. The main outcome measure was the midterm survival up to five years and the incidence of major adverse cardiovascular events (MACE) up to three years.

**Results:**

Since April 2002, one hundred consecutive patients underwent either off pump robotic assisted single vessel small thoracotomy (SVST, n = 88), or off pump total endoscopic coronary artery bypass grafting (TCAB, n = 12). All patients were operated on by the same primary surgeon but different assisting surgeons. All patients received a left internal mammary arterial (LIMA) graft as planned. The primary outcome of total one month and three years MACE and up to five year survival was 0, 9 and 96% respectively.

**Conclusions:**

The procedural success rates in terms of morbidity and mortality up to five years are compatible to the outcomes observed outside the United Kingdom. These results are not inferior to that of conventional off pump single vessel coronary surgery or percutaneous coronary intervention to the LAD.

## Background

**Figure 1:**
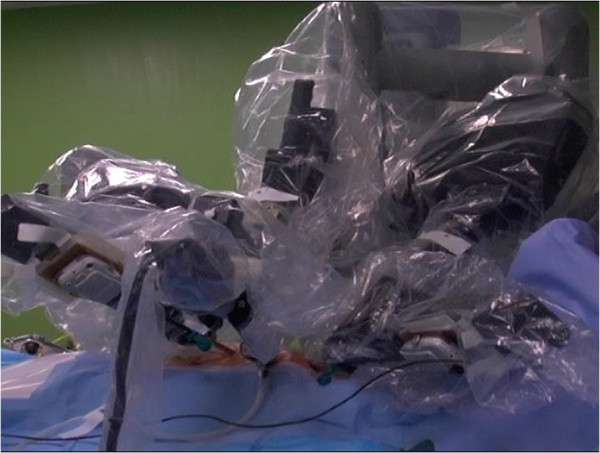
**The standard second generation Da Vinci robot with three robotic arms.** The middle arm commonly used for the camera.

In 1988, a robot developed at The Imperial College, London was used to perform prostate surgery. This was one of the first prototypes of its kind. The first reported case of closed chest coronary surgery using the Da Vinci robot (Intuitive Surgical, USA) took place at the turn of the century by Utz Kappert [1]. Since our first reports of successful attempts at robotically assisted coronary revascularisation back at imperial college [2,3], over one hundred coronary procedures have been successfully performed using the same second generation Da Vinci robot (Figure 1). In 2010 we published the results of a feasibility study and our short term outcomes in the same group of patients [4]. In the current study in accordance to the definition, and the principles of best practice in clinical audit [5,6], we compared our midterm outcomes of mortality and major adverse cardiovascular events (MACE) against international literature.

## Methods

This is an Institutional audit. We investigated our midterm outcomes in terms of MACE up to three years and mortality for up to five years for single vessel small thoractomy (SVST) and total endoscopic coronary bypass grafting (TECAB). Permission was granted by the Imperial Collage Healthcare Trust audit committee and information was obtained retrospectively from patients records, communication with their primary care physician and the National Strategic Tracing Service. Patients had undergone either SVST or TECAB. All other robotic or other non robotic minimal invasive coronary revascularisations such as MIDCAB (Minimal invasive direct coronary artery bypass) were excluded. The primary end points were the midterm up to five years survival and major adverse cardiovascular event such as death, myocardial infarction (MI), stroke and the need for target vessel revascularisation (TVR). The secondary end points were conversion rates to MIDCAB, re-operation for bleeding, the incidence of atrial fibrillation, respiratory complications and the median length of stay in hospital.

**Figure 2:**
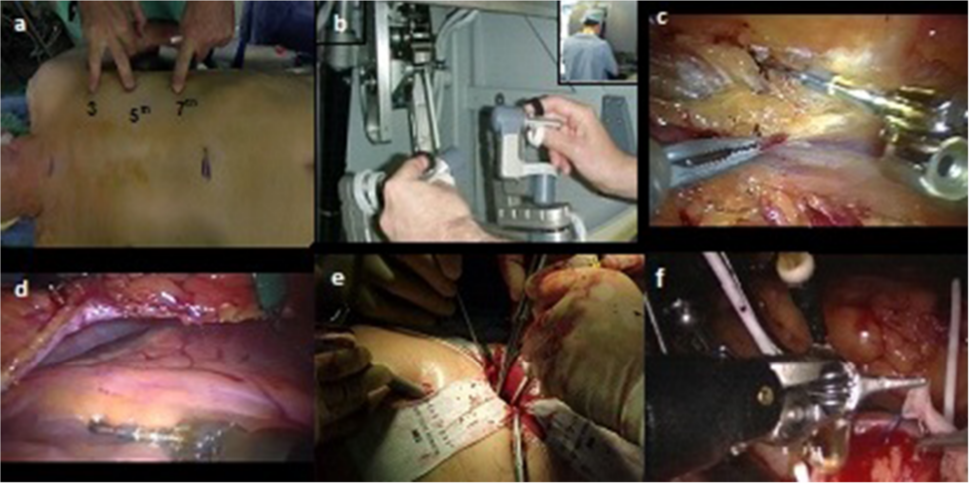
**SVST/TECAB surgical sequence. a)** The port access position in the third, fifth and seventh interspaces. **b)** The operating surgeon controls the robotic arms from the console. **c)** The Left internal mammary artery is harvested using the Da Vinci robot. **d)** Pericardotomy is performed to expose the targeted left anterior descending artery. Then the coronary anastomosis is performed either **e)** through a small left thoracotomy (SVST) or **f)** totally endoscopically (TECAB).

All patients signed an informed consent form prior to their operative procedure and were fully aware of their surgical approach options. All procedures were performed by the same principal surgeon. A second generation standard da Vinci robot was used in all cases. The robotic arms gained access into the left thoracic cavity through three ports. First the left internal mammary artery (LIMA) was harvested and the left anterior descending artery (LAD) exposed through a pericardotomy. The LIMA to LAD anastomosis was performed either through a small anterior thoracotomy in SVST or it was performed totally endoscopically using the robotic arms in TECAB (Figure 2a-f).

## Results

One hundred patients fitted our criteria (SVST, n = 88 & TECAB, n = 12). Table 1a summarises the patient characteristics. There were striking features such as male predominance and a raised body mass index (BMI). Patients were otherwise not discriminated on the basis of being diabetic, having carotid disease or poor left ventricular function. One in ten patients had three vessel coronary artery disease and one in five had their robotic procedure as part of a hybrid strategy which consisted of an additional percutaneous coronary intervention (PCI). The majority of patients had type B or C lesion characteristics which according to the American Heart Association (AHA) guidelines [7] places them at a moderate to high risk, with moderate to low chance of a successful PCI.

**Table 1:** Summary of patient characteristics and outcomes

a) Preoperative characteristics		b) Primary outcome measures	
Number of patients in total	100	Peri-operative MACE (%)	0
Age	62 ± 11	Survival to discharge (%)	100
Male	95	30 Day MACE (%)	0
BMI Kg/m^2^	27.0 ± 3.3	Three year MACE (%)	9
Diabetes (%)	17	Three year Incidence of Death (%)	3
Mean creatinine	100 ± 22	Three year Incidence of MI (%)	2
Carotid disease (%)	10	Three year Incidence of Stroke (%)	1
Previous MI (%)	18	Three year Incidence of TVR (%)	3
Poor LV (%)	1	Five year incidence of death (%)	4
Previous PCI (%)	13		
Hybrid strategy (%)	19	c) Secondary outcome measures	
Redo (%)	1	Conversion rate to MIDCAB (%)	3
EUROScore	1.73 ± 1.93	Re-operation for bleeding (%)	0
NYHA (III&IV) (%)	18	Atrial fibrillation (%)	2
CCS (III&IV) (%)	18	Respiratory complications (%)	4
Lesion charachteristic		Median length of stay (days)	4 ± 1
Type A (%)	10		
Type B (%)	55		
Type C (%)	35		

There were no major peri-operative cardiovascular events. There were no conversions to median sternotomy or a need for cardiopulmonary bypass. All patients survived to discharge. The primary outcome defined as the total MACE was 0% at one and twelve months and 9% after three years. The five year mortality excluding the patients operated less than 5 years from this analysis was 4% (n = 85). Table 1b illustrates the 3 year MACE split into its components of death 3%, myocardial infarction 2%, stroke 1% and target vessel revascularisation (TVR) in 3%. A number of secondary outcome measures were also observed (Table 1c), such as the conversion rate to MIDCAB 3%, incidence of atrial fibrillation (AF) 2%, respiratory complications such as post operative left sided pleural effusion in 4% and a median length of stay in hospital of 4 ± 1 days.

## Discussion

This is an audit into the primary and secondary outcomes following robotic assisted single vessel coronary surgery including short and medium term mortality, the incidence of major adverse cardiovascular complications and five year survival. The subjects formed the largest cohort of patients in the United Kingdom who had their coronary revascularisation enhanced using the Da Vinci robot.

The major limitation of this study is that there was no compatible local control group to compare this cohort against. The majority of patients who presented with a single vessel LAD disease were considered and accepted for robotic assisted surgery. A small number of female patients with upper body obesity (large breasts) or patients with chest wall deformities were not considered as candidates for this procedure. They were treated by the conventional off pump method; however their number was too small to be used as a control.

This audit was also limited due to its retrospective observational nature and that it lacks long term results. A prospective or a propensity matched analysis would improve the strength of these conclusions. However in this cohort neither was possible. This study started over half way through the patient recruitment process and the population was grossly skewed with the male gender being over selected for the reasons already mentioned such as the upper body obesity and chest deformity. With respect to long term outcomes, this cohort is still under observation and will be re audited with respect to their long term outcomes.

In addition there were a number of procedure specific limitations to the use of robotic enhanced coronary grafting. One of the major limitations was the lack of fourth arm on our early generation robot. This made it technically challenging to perform the total endoscopic procedures as well as extending the target anastomosis to any other than the LIMA to the LAD. In order to perform our totally endoscopic cases in this part of our experience we used an externally operated mechanical suction stabiliser inserted via a 4th thoraco-port. The latest generation of Da Vinci robot with a fourth arm allows the use of a suction stabiliser which is console-driven instead. In addition bulkiness of the instruments also made this approach unsuitable for smaller patients or ones with chest wall deformities. This in a number of cases resulted in leverage for example against a protruding shoulder. These problems may be resolved by development and innovation of bioinspired and flexible robotic systems such as the i-Snake® bimanual robot (Hamlyn Centre, Imperial Collage, London).

We used a benchmarking approach to report our national experience be it from a single centre. As there are no national standards for this type of surgery, we have used the available worldwide literature as the standard against which we conducted our audit.

Our results indicate that the primary outcomes matched that of the literature for conventional single vessel off pump coronary artery bypass (OPCAB) to the LAD in terms of short and medium term survival. These have been illustrated in Table 2. The reported thirty days and 12 month mortalities according to Herz et al. were 1% and re intervention rates were 2% with conventional off pump LIMA to LAD single graft anastomoisis as compared to our 0% thirty days and twelve months mortality and 3% at 3 years [8]. Halkos et al. compared sternotomy versus non-sternotomy LIMA to LAD grafting using either minimally invasive or robotic assisted harvesting of the LIMA, followed by direct anastomosis to the LAD through a small anterolateral thoracotomy. All 597 patients had single vessel coronary disease. In their propensity adjusted comparison, sternal sparing incisions were associated with insignificant differences in 30 day adverse events such as mortality 1.1% versus 0.9%, myocardial infarction 1.4% versus 0.4% and 2.2 versus 2.6% for total MACE. Whereas the incidence of stroke was higher in sternotomy group 0% versus 1.3% for non-sternotomy versus sternotomy respectively (p = 0.03) [9]. Our incidence of Stroke was only 1% at three years. Kapper et al. Reported their five year follow-up of robotic endoscopic coronary artery bypass. They reported on 41 patients with proximal LAD disease who had TECAB. 33 of these patients had the procedure on a beating heart and of that 30 patients had single LIMA to LAD anastomosis using the Da Vinci robot. The rest (n = 11) had either on pump or multi vessel bypass. Their overall five year survival was 92.7% however their freedom from major cardiovascular event at five years was 75.6% [10]. The combined experience described by Bonatti et al. of 498 patients who had on-pump single and multi vessel TECAB showed a five year survival of 95.0% [11]. Their experience with on-pump single vessel TECAB in 334 patients revealed a five year survival of 95.8% and a freedom from MACE at five years of 73.5% [12]. All of our twelve off-pump TECAB patients are alive over five years from their surgery. However it is not possible to make a fair comparison between our outcomes in this category (TECAB) compared to the experience of Bonatti or Kapper.

**Table 2:** Comparison of composite outcomes in the published literature

**Study name**	**Single vessel LAD revascularisation**	**Number of patients**	**Peri-operative MACE**	**Secondary outcomes**	**30 day MACE**	**12 month MACE**	**18 +/- 16/12 MACE**	**3 year MACE**	**Survival at five year**
Herz et al.	Conventional OPCAB using LIMA	386		2.8% Bleeding		1% Mortality			
				8.5% AF					
				6 days MLOHS		2% TVR			
Vassiliades et al.	Endo-ACAB using LIMA	607	1% Mortality	1.6% Bleeding			2% Mortality		92%
			1.6% MI	19.6% AF			2.1% MI		
			0.3% Stroke	11 days MLOHS			0.8% Stroke		
			1.5% TVR	3.6% Conversion to sternotomy			0.7% TVR		
				9% Respiratory complications					
Kappert et al.	TECAB	41							92.7%
Kapoor et al.	PCI vs. Standard CABG using LIMA	633 vs. 577			1% Mortality for both	0.3 vs. 2.1% Mortality			92.8 vs. 90.6%
						19.5 vs. 4% TVR			
Bonatti et al.	TECAB	334	0.3% Mortality						95.8%
Halkos et al.	Sternotomy vs. Minimal invasive Non-sternotomy using LIMA	234 vs. 363			1.1 vs. 0.9% Mortality				
					1.4 vs. 0.4% MI				
					0 vs. 1.3% Stroke				
					2.2 vs. 2.6% MACE				
Casula et al.	Robotic enhanced using LIMA	100	0% Mortality	0% Bleeding	0% Mortality	0% Mortality		3% Mortality	96%
				2% AF					
			0% MI	4 days	0% MI			2%	
			0% Stroke	MLOHS	0% Stroke			MI	
				3.0%	0% TVR			1% Stroke	
			0% TVR	Conversion to					
				MIDCAB				3% TVR	
				4% Respiratory complications					

*MACE* Major adverse cardiovascular events, *MLOHS* Mean length of hospital stay, *TVR* Target vessel revascularisation, *AF* Atrial fibrillation, *MI* Myocardial Infarction, *LIMA* Left internal mammary artery, *OPCAB* Off pump coronary artery bypass, *CABG* Coronary artery bypass graft, *TECAB* Totally endoscopic coronary artery bypass, *Endo-ACAB* Endoscopic atraumatic coronary artery bypass, *PCI* Percutanious coronary intervention, *LAD* Left anterior descending.

The long term results of endoscopically harvested LIMA followed by atraumatic coronary artery bypass (endo-ACAB) of LIMA to LAD was reported by Vassilades et al. In a retrospective study he reviewed 607 consecutive patients that underwent endo-ACAB over eight years [13]. They reported a 30 day mortality of 1% and an overall angiographic patency of over 95% for all lesion characteristics. At five years they showed an event free survival of 92% (See Table 2 for breakdown of MACE). The event free survival for our cohort at three years was 91%.

Our secondary outcomes were better than reported outcomes in a similar cohort [14]. In a robotically-assisted off pump coronary revascularisation study through a small anterior thoracotomy, Turner et al. reported a 2.8% incidence of reoperation for bleeding, atrial fibrillation (AF) occurred in 8.5% of patients and the average length of stay in hospital was 5.7 days compared to our report of 0%, 2% and 4 days respectively. They also reported a similar 0% operative mortality.

In a meta-analysis of the randomized trials [15] Kapoor et al. concluded that in patients with single vessel proximal LAD disease, who were eligible for both procedures there was no difference in survival at any key time points between PCI and CABG (99% for both by 30 days, 97.9% at one and 92.8% at five years with CABG versus 99.7% at one and 90.6% at five years for PCI), nor were there any difference in the rate of stroke or myocardial infarction, but a significantly lower incidence of repeated revascularisation in the CABG group (4% versus 19.5% at one year).

Obesity is not an independent risk factor for poor outcome in minimally invasive cardiac surgery [16]. Being overweight did not necessarily make this operation more difficult [4]. However as the robotic arms are relatively cumbersome the shape of the patient becomes a limiting factor. For example Small female patients with relatively large breasts were excluded on the basis of their size and to avoid placing the robotic arms through the female breast tissue. Patients with a deformed chest were also considered carefully. The majority of the subjects in this audit were essentially overweight men.

All the patients in this cohort received an arterial (LIMA) graft. This is 100% arterial revascularisation compared to 85% of patients who were destined for a single vessel CABG through a standard median sternotomy according to the sixth national adult cardiac surgery database.

Finally although there is evidence supporting the use of the robot to improve post operative pain, general health and physical function scores as compared to conventional sternotomy [11], these conclusions have been drawn from observational studies. We reported our anecdotal evidence in favour of robotic enhancement through earlier return to activities of daily living in our last publication [4]. However there have been no randomised trials in this subject.

## Conclusion

These results show compatible national outcomes for robotically enhanced coronary surgery in terms of midterm MACE and up to five years survival in the United Kingdom. They suggest similar outcomes to single vessel CABG or OPCAB through standard sternotomy, endo-ACAB as well as percutaneous coronary interventions and similar practices of robotically enhanced coronary procedures elsewhere in the world.

## Abbreviations

AF: Atrial fibrillation; AHA: American Heart Association; BMI: Body mass index; CABG: Coronary artery bypass grafts; Endo-ACAB: Endoscopic atraumatic coronary artery bypass; LAD: Left anterior descending artery; LIMA: Left internal mammary artery; MACE: Major adverse cardiovascular event; MI: Myocardial infarction; MIDCAB: Minimal invasive coronary artery bypass; MLOHS: Mean length of hospital stay; OPCAB: Off pump coronary artery bypass; PCI: Percutanious coronary intervention; SVST: Single vessel small thoracotomy; TECAB: Total endoscopic coronary artery bypass; TVR: Target vessel revascularisation.

## Competing interests

The authors declare that they have no competing interests.

## Authors’ contributions

RC has made substantial contribution to the conception, acquisition of data, data analysis, interpretation and critical revision of the manuscript for important intellectual content. EK has made substantial contribution to the study design, acquisition of data, data analysis, interpretation, drafting the manuscript revising it critically for important intellectual content. He is the corresponding author. TA has made substantial contribution to the study design, data analysis, interpretation and critical revision of the manuscript for important intellectual content. All authors read and approved the final manuscript.

## Authors’ information

First author: Roberto Casula, Department of Cardiothoracic Surgery. Hammersmith Hospital, Shepherds Bush, London, England, W12 0HS.

Corresponding author: Espeed Khoshbin, University Hospital of Central Manchester, Oxford Road, Manchester M13 9WL, UK.

Last author: Thanos Athanasiou, The Department of Biosurgery and Surgical Technology-Imperial College London10th Floor, Queen Elizabeth the Queen Mother (QEQM) Building St Mary’s Hospital Campus Praed Street, London, W2 1NY.

Presentation: Oral presentation at the Royal Society of Medicine, 29th of June 2012. Through the keyhole: Where we are with minimally invasive cardiothoracic surgery.
